# Fillet of pinna flap in scalp reconstruction

**DOI:** 10.1186/1471-2482-13-S1-A45

**Published:** 2013-09-16

**Authors:** Luigi Esposito, Mariagrazia Moio, Ilaria Mataro, Bianca Aceto, Elena Ambrosino, Sergio Razzano, Fabrizio Schonauer

**Affiliations:** 1 Plastic Surgery Unit, University Federico II – Via Sergio Pansini 5, Naples, Italy

## Introduction

Fillet flaps are commonly used to cover skin defects after trauma or tumors, especially in the extremities [1-5].

## Methods

We report four patients operated in the last three years, for lesions of the ear, invading the post auricular skin and infiltrating into the scalp.

On examination these ulcerating lesions had the clinical appearance of deeply infiltrating basal cell carcinoma. Surgery was performed under general anaesthesia.

After resection was performed with 1 cm skin margins, down and including the temporal fascia, the clear anterior skin surface of the upper pole of the ear was freed by its internal cartilaginous skeleton. The obtained fillet flap of the homolateral upper ear pole was raised and reflected posteriorly to cover the wound completely or partially. When needed, a split skin graft was added to the reconstruction.

## Results

Histology gave the confirmation of the diagnosis and indications on the radicality in the operated patients. The cosmetic result was satisfactory. Follow up at a mean 6 months showed no recurrence.

## Conclusions

Several possibilities for the coverage of scalp defects have been reported[6], including local or pedicled flaps.

We describe the use of fillet of pinna flap to cover periauricular scalp defects. We are not aware of any report of this type of flap to cover defects following tumour resection.

The main advantages of the fillet of pinna flap are its durable coverage, colour match of the skin and absence of a donor site defect. In our patients, flaps were composed of healthy tissue which otherwise would have been discarded in the resection. In our opinion, fillet of pinna flaps can be used to cover the periauricular scalp defects when the auricular skin is clear and not been invaded by the lesion.
